# Insights into the Multi-Dimensional Dynamic Landscape of Epithelial–Mesenchymal Plasticity through Inter-Disciplinary Approaches

**DOI:** 10.3390/jcm9061624

**Published:** 2020-05-27

**Authors:** Mohit Kumar Jolly, Toni Celia-Terrassa

**Affiliations:** 1 Centre for BioSystems Science and Engineering, Indian Institute of Science, Bangalore 560012, India; 2 Cancer Research Program, IMIM (Hospital del Mar Medical Research Institute), 08003 Barcelona, Spain

Epithelial–mesenchymal transition (EMT), first described by Dr. Elizabeth (Betty) Hay in the 1980s during vertebrate embryonic development [1], has important implications in cancer aggressiveness [2]. EMT is a cellular biological process in which cells lose their epithelial characteristics such as cell polarity and cell–cell adhesion and gain traits of the mesenchymal phenotype such as invasive and migratory abilities [3]. Recent in vitro, in vivo and in silico investigations have highlighted that EMT is a continuum of many hybrid states with various combinations of epithelial and mesenchymal markers and traits, associated with high phenotypic plasticity, demonstrated by dynamic reversible transitions as well as the gain of stemness, drug resistance and metabolic adaptability [4,5]. This revised perspective has led to EMT and its reverse process mesenchymal to epithelial transition (MET) being referred to as epithelial–mesenchymal plasticity (EMP). Classically, the EMP status of a cell has been characterized by molecular markers associated with epithelial (E-cadherin and cytokeratin) and mesenchymal (vimentin, N-cadherin and fibronectin) phenotypes. However, EMP characterization based only on molecular markers is not sufficient. Thus, new recent methods of characterizing EMP based on cellular morphology, biophysical traits, and functional properties (such as response to drugs) has been proven to be useful to understand EMP from a multi-dimensional dynamic perspective.

In this Special Issue, the authors discuss the implications of the EMP seen in stemness, drug resistance [6], immune-suppression [7,8,9], metabolic reprograming and the interaction of cancer cells with the tumor microenvironment. We have diverse topics related to the transcriptional and microRNA-mediated control of EMP networks [10,11], the multi-omics [12,13] and morphological mapping of EMP [14], integrative approaches to link EMP with metabolic reprogramming and/or autophagy [15,16], and the designing of novel ex vivo systems such as microfluidic setups and 3D polymer scaffolds to visualize the dynamics and heterogeneity of EMP in cancer cells [17,18]. This Special Issue, through a collection of review and research articles, represents the emerging inter-disciplinary approaches being taken to elucidate the nonlinear dynamics of EMP and their differential contributions to disease aggressiveness.

Phenotypic plasticity can often lead to non-mutational heterogeneity, thus further increasing the “fitness” of cells in dynamic environments [5]. Such heterogeneity was seen in EMP as well in a study with single cell-derived clonal progenies from established subpopulations in PMC42 breast cancer cells. These progenies showed clonal diversity and intrinsic plasticity with varied functional traits such as proliferation, stemness, therapy response, migration and invasion [19]. The migration and invasion traits are not only cell-autonomous; instead, they can be affected by the effects of cancer cells on the extracellular matrix (ECM) by degrading ECM proteins. Singh et al. showed how increased dermatan sulphate (DS) in breast cancer cells can facilitate invasion and morphological changes by remodeling the fibrillar matrix microenvironment [20]. Thus, cancer cells exhibiting varying degrees of EMP may exhibit individual and/or cooperative cell migration; different relative levels of biomarkers for these different migration modes were shown to be associated with aggressive cancer progression [16,21].

EMP has also been shown to cross paths with metabolic reprograming—an important hallmark of cancer [4]. Here, Liu et al. showed how Snail—a well-known EMT-inducer—can reduce the oxidative metabolism of pancreatic cancer cells and increase glucose uptake and lactate production, thus increasing glucose metabolism [22]. In another study by Huang et al. in colorectal cancer, ATP synthase subunit ε was found to be upregulated and promote metastasis by inducing EMT [23].

Overall, this collection of articles represents the diversity of experimental and computational tools and approaches, academic backgrounds and contributions that have been made by experts who have contributed to our understanding of the mechanisms and implications of EMP in cancer progression. We sincerely hope that this collection will serve as a valuable resource, enabling the cross-fertilization of ideas, with the goal of characterizing the dynamics of EMP and their contribution to disease burden.
